# The FibroScan-aspartate aminotransferase score can stratify the disease severity in a Japanese cohort with fatty liver diseases

**DOI:** 10.1038/s41598-021-93435-x

**Published:** 2021-07-05

**Authors:** Hideki Fujii, Shinya Fukumoto, Masaru Enomoto, Sawako Uchida-Kobayashi, Tatsuo Kimura, Akihiro Tamori, Yuji Nadatani, Shingo Takashima, Naoki Nishimoto, Norifumi Kawada

**Affiliations:** 1 Department of Premier Preventive Medicine, Graduate School of Medicine, Osaka City University, 1-4-3, Asahimachi, Abeno, Osaka, 545–8585 Japan; 2 Department of Hepatology, Graduate School of Medicine, Osaka City University, Osaka, Japan; 3 Division of Data Management, Division of Biostatistics, Clinical Research and Medical Innovation Center, Hokkaido University Hospital, Sapporo, Japan

**Keywords:** Non-alcoholic fatty liver disease, Laboratory techniques and procedures

## Abstract

This study aimed to prove that the FibroScan-aspartate aminotransferase (FAST) scores can be used to stratify disease severity in a Japanese cohort with fatty liver diseases [metabolic dysfunction-associated fatty liver disease (MAFLD) and nonalcoholic fatty liver disease (NAFLD)]. All the participants (n = 2254) underwent liver stiffness measurements and controlled attenuation parameter assessments. We compared the clinical characteristics of the patients with MAFLD and NAFLD using the FAST scores and explored the independent determinants of FAST scores ≥ 0.35, which indicated possible progressive disease. Overall, MAFLD was diagnosed in 789 patients (35.0%), while NAFLD was diagnosed in 618 (27.4%). The proportion of patients that had a condition that suggested progressive liver disease was higher in those with MAFLD than in those with NAFLD [68 (8.6%) vs 48 (7.7%)]. The area under the receiver-operating characteristic curve of the FAST score for diagnosing advanced fibrosis was 0.969 in MAFLD and 0.965 in NAFLD. Multivariate analyses determined that diabetes mellitus, alanine aminotransferase (ALT) levels, fatty liver index, and Fibrosis-4 index independently predict FAST scores ≥ 0.35 in patients with MAFLD. ALT levels had the strongest correlation with the FAST scores (p = 0.7817). The FAST score could stratify the disease severity in the Japanese cohort with fatty liver diseases.

## Introduction

Because of widespread obesity, nonalcoholic fatty liver disease (NAFLD) is one of the leading liver diseases worldwide. The global prevalence of NAFLD is currently estimated to be 25%^1^. NAFLD comprises a broad spectrum of diseases ranging from nonalcoholic fatty liver disease to nonalcoholic steatohepatitis (NASH), and it can progress to cirrhosis and/or hepatocellular carcinoma^2,3^. Although most cases of NAFLD do not progress to advanced fibrosis or cirrhosis, the high prevalence of NAFLD implies that many patients develop chronic liver disease. Furthermore, NAFLD is one of the main indications for liver transplantation in Europe^4^ and the United States^5^. In 2020, an international consensus panel suggested that NAFLD should be redefined as metabolic dysfunction-associated fatty liver disease (MAFLD) and proposed new information regarding its diagnosis^6,7^.

Currently, no pharmacotherapies for NASH have been approved by the Food and Drug Administration or European Medicines Agency, and various clinical trials are ongoing^8,9^. Therefore, it has become increasingly important to identify patients with NASH who are at risk of progression to cirrhosis. The FibroScan-aspartate aminotransferase (FAST) score is a simple algorithm that can diagnose NASH using an elevated (≥ 4) NAFLD activity score (NAS) and significant fibrosis score (≥ 2)^10^. The FAST score is determined by the liver stiffness measurement (LSM) obtained using vibration-controlled transient elastography (VCTE), estimation of the controlled attenuation parameter (CAP) obtained using a FibroScan device, and estimation of the aspartate aminotransferase (AST) level. The FAST score is expected to reduce unnecessary liver biopsies performed for patients unlikely to have significant disease^10^. However, there have been no reports on the stratification of disease severity using FAST-score in the patients with MAFLD and NAFLD in the general population. The aim of this study was to prove that FAST-score can be used to stratify disease severity of fatty liver diseases (MAFLD and NAFLD), and to determine the clinical differences between the two using a general population cohort. We also examined which factors available in routine clinical practice affect the FAST score. This is the first study to examine the clinical implications of the FAST score in a general population.

## Results

### Baseline characteristics of patients

**Figure 1:**
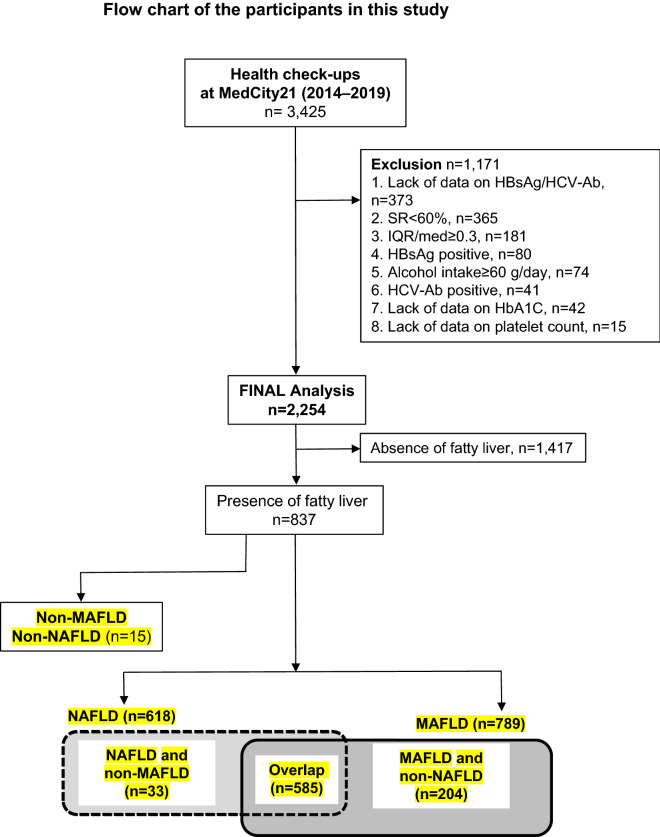
Flow chart of the study participants. HbA1C: glycated hemoglobin; HBV: hepatitis B virus; HBsAg/HCV-Ab: hepatitis B surface antigen/anti-hepatitis C antibody; HCV: hepatitis C virus; IQR/med: interquartile range/median; NAFLD: nonalcoholic fatty liver disease; MAFLD: metabolic dysfunction-associated fatty liver disease.

The study population comprised 2254 participants who fulfilled the inclusion criteria (Fig. 1). Among these participants, MAFLD was diagnosed in 789 individuals (35.0%), while NAFLD was diagnosed in 618 (27.4%) of the overall population. A total of 585 (26.0%) cases met both MAFLD and NAFLD definition criteria (overlap group). A total of 15 cases did not meet both MAFLD and NAFLD definition criteria (Non-MAFLD Non-NAFLD group). The clinical characteristics of the patients with MAFLD, non-MAFLD, and NAFLD are shown in Table 1. The median age of patients with MAFLD and NAFLD were 53 and 53 years, respectively, and 74% and 68% of the patients were male, respectively. The median body mass index (BMI) of patients with MAFLD and NAFLD was 25.4 and 25.3 kg/m^2^, and diabetes mellitus (DM) was present in 23% and 21% of these patients, respectively. Using the FAST score, 9 (1.1%) and 6 (1.0%) patients with MAFLD and NAFLD were at high-risk and 59 (7.5%) and 42 (6.7%) were at intermediate risk for progressive liver disease, respectively. Therefore, approximately 9% and 8% of patients with MAFLD and NAFLD who underwent health examinations had a condition that could not be ruled out as progressive liver disease. We also analyzed the clinical characteristics of fatty liver in detail (Supplementary Table 1). The patients with NAFLD and non-MAFLD were significantly younger than the overlap group and older than the MAFLD and non-NAFLD group. Fatty liver index (FLI) and FAST-score in the patients with NAFLD and non-MAFLD were significantly lower than those in the overlap and MAFLD and non-NAFLD group.

**Table 1:** Clinical characteristics of patients with MAFLD and NAFLD.

Variables	Non-MAFLD (n = 1465)	MAFLD (n = 789)	NAFLD (n = 618)
Age (years)^a^	52 (43–62)	53 (46–63)	53 (46–63)
Sex (male)^b^	721 (51)	586 (74)	423 (68)
BMI (kg/m^2^)^a^	21.8 (20.1–23.6)	25.4 (23.8–27.3)	25.3 (23.5–27.1)
WC (cm)^a^	81.0 (75.5–85.6)	91.0 (86.8–96.1)	91.0 (86.1–96.0)
Alcohol intake (g/day)^a^	4.3 (0.14–21.4)	4.3 (0.1–25.7)	0.14 (0.14–10)
DM^b^	88 (9)	178 (23)	132 (21)
Hypertension^b^	303 (21)	310 (39)	223 (36)
Dyslipidemia^b^	563 (38)	609 (77)	470 (76)
Total bilirubin (mg/dL)^a^	0.7 (0.6–1.0)	0.7 (0.6–0.9)	0.7 (0.6–0.9)
AST (U/L)^a^	19 (17–24)	23 (19–28)	22 (18–27)
ALT (U/L)^a^	16 (13–22)	26 (19–40)	26 (19–39)
GGT (U/L)^a^	20 (14–32)	38 (25–58)	33 (23–50)
Uric acid (mg/dL)^a^	5.2 (4.3–6.1)	6.4 (5.4–7.2)	6.2 (5.2–7.1)
Albumin (g/dL)^a^	4.3 (4.1–4.4)	4.3 (4.2–4.5)	4.4 (4.2–4.5)
Platelet count (× 10^9^/L)^a^	221 (192–254)	229 (193–264)	230 (195–264)
Total cholesterol (mg/dL)^a^	199 (178–222)	202 (179–225)	201 (178–226)
TG (mg/dL)^a^	75 (56–103)	129 (95–187)	123 (92–175)
HDL-cholesterol (mg/dL)^a^	60 (49–71)	46 (38–54.5)	46 (38–54)
LDL-cholesterol (mg/dL)^a^	113 (95–135)	123 (101–144)	126 (102–145)
Non-HDL cholesterol (mg/dL)^a^	137 (117–159)	155 (134–176)	156 (133–176)
Fasting plasma glucose (mg/dL)^a^	99 (94–105)	108 (101–119)	107 (99–117)
HbA1C (%)^a^	5.6 (5.4–5.8)	5.8 (5.6–6.1)	5.8 (5.6–6.1)
**NIT**			
FLI^a^	10.9 (4.9–22.9)	48.1 (31.3–70.4)	43.4 (28.0–66.0)
FIB-4^a^	1.09 (0.81–1.55)	1.03 (0.76–1.39)	0.99 (0.73–1.34)
NFS^a^	− 2.08 (− 2.79 to − 1.25)	− 1.76 (− 2.60 to − 0.91)	− 1.88 (− 2.76 to − 0.98)
**VCTE**			
LSM^a^	3.6 (3.0–4.3)	4.2 (3.5–5.0)	4.1 (3.5–4.9)
CAP^a^	216 (189–245)	288 (255–318)	289 (256–318)
**FAST score**	0.04 (0.02–0.07)	0.09 (0.05–0.18)	0.09 (0.04–0.16)
< 0.35^b^	1456 (99.4%)	721 (91.4%)	570 (92.3%)
0.35–0.65^b^	9 (0.6%)	59 (7.5%)	42 (6.7%)
> 0.67^b^	0 (0%)	9 (1.1%)	6 (1.0%)

ALT: alanine aminotransferase; AST: aspartate aminotransferase; BMI: body mass index; CAP, controlled attenuation parameter; DM: diabetes mellitus; FAST: FibroScan-aspartate aminotransferase; FIB-4: fibrosis-4; FLD: fatty liver disease; FLI: fatty liver index; GGT: gamma-glutamyltransferase; HDL: high-density lipoprotein; LDL: low-density lipoprotein; LSM, liver stiffness measurement; MAFLD: metabolic dysfunction-associated fatty liver disease ; NAFLD: nonalcoholic fatty liver disease; NFS: NAFLD fibrosis score; NIT: noninvasive test; TG: triglycerides; VCTE: vibration controlled transient elastography; WC: waist circumference.

^a^Median (interquartile range), ^b^number (%).

### Diagnostic abilities of FAST score, fatty liver index, CAP, and BMI for fatty liver in patients with MAFLD and NAFLD

We investigated the diagnostic abilities of FAST, FLI, CAP, and BMI for fatty liver in patients with MAFLD and NAFLD (Supplementary Figure 1). The area under the receiver-operating characteristic (AUROC) curve for diagnosing MAFLD was largest for FLI (0.882), followed by CAP (0.867), BMI (0.849), and the FAST score (0.753). The AUROC for diagnosing NAFLD was largest for CAP (0.835), followed by FLI (0.792), BMI (0.788), and the FAST score (0.693). The optimal cut-off of hepatic steatosis according to CAP was 259 dB/m in patients with MAFLD and 258 dB/m in patients with NAFLD.

### Diagnostic abilities of the FAST score, FIB-4, and NAFLD fibrosis score (NFS) for significant fibrosis in patients with MAFLD and NAFLD

Next, we investigated the diagnostic abilities of the FAST score, FIB-4, and NFS for significant fibrosis in patients with MAFLD and NAFLD (Supplementary Figure 2). The AUROC curve for diagnosing significant fibrosis in patients with MAFLD was largest for the FAST score (0.890), followed by NFS (0.585) and FIB-4 (0.549). The AUROC curve for diagnosing significant fibrosis in patients with NAFLD was the largest for the FAST score (0.888), followed by the NFS (0.561) and FIB-4 (0.510). We also investigated the diagnostic ability of FAST score for advanced fibrosis in patients with MAFLD and NAFLD (Supplementary Figure 3). The AUROC curve for diagnosing advanced fibrosis was 0.969 in MAFLD and 0.965 in NAFLD.

### Comparisons of the clinical characteristics of patients with MAFLD using the FAST score

Next, we investigated the clinical characteristics of MAFLD using the FAST score (Table 2). Stepwise increments in the prevalence of DM, AST levels, alanine aminotransferase (ALT) levels, gamma-glutamyl transferase (GGT) levels, fasting plasma glucose, HbA1c levels, FIB-4, and LSM were observed.

**Table 2:** Comparison of the clinical characteristics of MAFLD patients by FAST score.

Variables	FAST score		
	< 0.35 (n = 721)	0.35–0.67 (n = 59)	> 0.67 (n = 9)
Age (years)^a^	54 (46–63)	51 (40–63)	54 (46–64)
Sex (male)^a^	528 (73)	51 (86)	7 (78)
BMI (kg/m^2^)^a^	25.3 (23.6–27.0)	27.2 (25.1–28.9)	26.8 (23.8–28.6)
WC (cm)^a^	91.0 (86.5–96.0)	94.5 (89.0–99.0)	93.0 (89.5–97.8)
Alcohol intake (g/day)^a^	4.3 (0.14–21.4)	10 (0.14–30)	21.4 (8.6–30)
DM^b^	146 (18)	26 (44)	6 (67)
Hypertension^b^	275 (38)	30 (51)	5 (56)
Dyslipidemia^b^	549 (76)	53 (90)	7 (76)
Total bilirubin (mg/dL)^a^	0.7 (0.6–0.9)	0.7 (0.6–0.9)	1.0 (0.7–1.3)
AST (U/L)^a^	22 (19–26)	43 (34–55)	79 (68–94)
ALT (U/L)^a^	25 (19–35)	69 (50–92)	124 (90–135)
GGT (U/L)^a^	36 (25–55)	64 (40–93)	121 (98–136)
Uric acid (mg/dL)^a^	6.3 (5.4–7.2)	7.0 (6.0–8.2)	5.0 (4.2–7.0)
Albumin (g/dL)^a^	4.3 (4.2–4.5)	4.4 (4.3–4.6)	4.3 (4.2–4.8)
Platelet count (× 10^9^/L)^a^	230 (195–265)	222 (189–264)	184 (144–220)
Total cholesterol (mg/dL)^a^	202 (180–224)	205 (177–237)	214 (163–236)
Triglycerides (mg/dL)^a^	125 (95–180)	170 (112–235)	153 (118–331)
HDL–cholesterol (mg/dL)^a^	46 (38.5–55)	41 (37–48)	44 (41–55)
LDL-cholesterol (mg/dL)^a^	124 (101–144)	118 (100–145)	118 (91–192)
Non-HDL cholesterol (mg/dL)^a^	155 (134–175)	160 (135–195)	167 (108–192)
Fasting plasma glucose (mg/dL)^a^	107 (101–118)	118 (109–137)	155 (105–184)
HbA1C (%)^a^	5.8 (5.6–6.1)	6.0 (5.8–6.6)	6.8 (5.9–7.7)
**NIT**			
FLI^a^	45.8 (30.4–67.5)	77.0 (52.2–88.9)	80.5 (59.5–87.0)
FIB-4^a^	1.03 (0.76–1.37)	1.08 (0.71–1.97)	2.08 (1.70–3.27)
NFS^a^	− 1.80 (− 2.59 to − 0.93)	− 1.61 (− 2.71 to − 0.56)	− 0.86 (− 1.56 − 0.09)
**VCTE**			
LSM^a^	4.1 (3.5 − 4.8)	6.0 (5.4–7.8)	10.0 (8.2–17.8)
CAP^a^	285 (252–311)	331 (307–359)	335 (312–360)

ALT: alanine aminotransferase; AST: aspartate aminotransferase; BMI: body mass index; CAP, controlled attenuation parameter; DM: diabetes mellitus; FAST: FibroScan-aspartate aminotransferase; FIB-4: fibrosis-4; FLI: fatty liver index; GGT: gamma-glutamyltransferase; HDL: high-density lipoprotein; LDL: low-density lipoprotein; LSM, liver stiffness measurement; MAFLD: metabolic dysfunction-associated fatty liver disease; NFS: NAFLD fibrosis score; NIT: noninvasive test; VCTE: vibration controlled transient elastography; WC: waist circumference.

^a^Median (interquartile range), ^b^number (%).

### Independent determinants associated with a FAST score ≥ 0.35 for patients with MAFLD

Table 3 presents the results of the independent determinants for a FAST score ≥ 0.35 and the condition that could not be ruled out as progressive liver disease for patients with MAFLD. During the univariate analyses, age, sex (male), BMI, waist circumference (WC), alcohol intake, presence of hypertension, presence of DM, presence of dyslipidemia, ALT level, FLI, FIB-4, and NFS were selected as significant variables. During the multivariate analyses, we selected age, sex (male), BMI, alcohol intake, presence of DM, ALT level, FLI, and FIB-4 as significant variables. The presence of DM, ALT level ≥ 42 U/L, FLI ≥ 63.9, and FIB-4 ≥ 1.75 were identified as independent determinants of a FAST score ≥ 0.35.

**Table 3:** Results of the univariate and multivariate analyses: explanatory variables for diagnosing a FAST score ≥ 0.35 in MAFLD patients.

Variables	Univariate analysis			Multivariate analysis		
	OR	*P* value	95% CI	OR	*P* value	95% CI
Age ≥ 45 years	0.39	0.0005	0.23–0.66	0.52	0.0929	0.24–1.11
Male sex	2.12	0.0331	1.06–4.23	0.97	0.9503	0.37–2.54
BMI ≥ 26.7 kg/m^2^	3.32	< 0.0001	2.00–5.51			
WC ≥ 90.5 cm	2.72	0.0005	1.54–4.80			
Alcohol intake ≥ 1.0 g/day	1.78	0.0354	1.04–3.03	1.53	0.2241	0.77–3.03
Presence of hypertension	1.72	0.0330	1.04–2.82			
Presence of DM	3.50	< 0.0001	2.10–5.83	3.85	0.0002	1.88–7.87
Presence of dyslipidemia	2.35	0.0270	1.10–5.01			
ALT ≥ 42 U/L	49.3	< 0.0001	20.9–116	45.1	< 0.0001	17.0–119.8
FLI ≥ 63.9	7.28	< 0.0001	4.15–12.8	2.39	0.0168	1.17–4.87
FIB-4 ≥ 1.75	4.19	< 0.0001	2.43–7.25	8.10	< 0.0001	3.34–19.6
NFS ≥ − 1.23	1.79	0.0223	1.09–2.96			

ALT: alanine aminotransferase; AST: aspartate aminotransferase; BMI: body mass index; DM: diabetes mellitus; FAST: FibroScan-aspartate aminotransferase; FLI: Fatty liver index; FIB-4: fibrosis-4; GGT: gamma-glutamyltransferase; HDL: high-density lipoprotein; MAFLD: metabolic dysfunction-associated fatty liver disease; NFS: NAFLD fibrosis score; WC: waist circumference.

### Correlations among FAST score, ALT level, and noninvasive test results for patients with MAFLD and NAFLD

**Figure 2:**
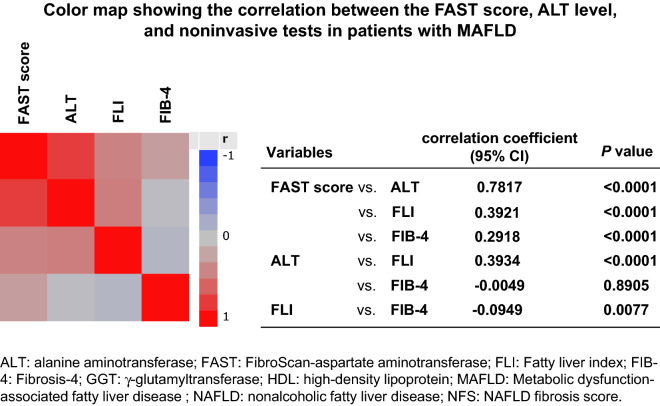
Color map showing the correlations among the FAST score, ALT level, and noninvasive test results for patients with MAFLD. ALT: alanine aminotransferase; FAST: FibroScan-aspartate aminotransferase; FLI: fatty liver index; FIB-4: Fibrosis-4; MAFLD: metabolic-associated fatty liver disease; CI: confidence interval.

We examined the correlations among the FAST score and ALT level (an index of inflammation), FLI (an index of hepatic steatosis), and FIB-4 (an index of hepatic fibrosis) in patients with MAFLD (Fig. 2). The correlation coefficient was highest for the ALT level (0.7817), followed by FLI (0.3921) and FIB-4 (0.2918). We also evaluated the correlations among the FAST scores and ALT levels, FLI and FIB-4 in patients with NAFLD (Supplementary Figure 4). The correlation coefficient was highest for the ALT level (0.7889), followed by FLI (0.3985) and FIB-4 (0.1632).

## Discussion

We found that approximately 9% and 8% of patients with MAFLD and NAFLD who underwent health examinations had conditions that could not be ruled out as progressive liver disease. Furthermore, noninvasive test results (FLI and FIB-4) and ALT levels were identified as independent determinants of progressive liver disease in patients with MAFLD.

Oeda et al. analyzed 166 patients with biopsy-proven NAFLD^11^ and found that approximately 25% of these patients fulfilled the histological criteria for progressive liver disease (NASH, NAS ≥ 4, and fibrosis score ≥ 2)^11^. Hofmann et al. reported the characteristics and demographics of an observational NAFLD cohort in Germany^12^. In the subset using the FAST score (n = 107), 16.8%, 34.6%, and 48.6% of the patients were classified as rule in NASH, gray zone, and rule out NASH, respectively^12^. Puri et al. investigated 199 United States veterans; among them, 29%, 36.5%, and 36.5% were classified as rule in NASH, gray zone, and rule out NASH, respectively^13^. A potential reason for the differences between the results of our study and those of other studies is the higher proportion of patients who underwent liver biopsy and had more progressive liver disease, thereby resulting in a referral bias. Another reason could be that most of the participants were healthy enough to attend work and were sufficiently health conscious to voluntarily undergo health examinations^14^, thereby resulting in a self-selection bias.

Newsome et al. studied the FAST score and reported that their method of diagnosing NASH with a NAS ≥ 4 and fibrosis score ≥ 2 was based on several therapeutic studies that revealed that the presence of increased necro-inflammatory activity was linked to progressive injury and the pharmacological response^10,15,16^. Therefore, we examined the interaction of each factor that comprises the FAST score (Table 3 and Fig. 2). We determined that all noninvasive factors, including steatosis (FLI), inflammation (ALT), and fibrosis (FIB-4), were independent determinants of progressive liver disease. In particular, the ALT level exhibited the strongest correlation with the FAST score. In the current medical system, many primary care centers and health examination centers do not have FibroScan devices. Therefore, MAFLD patients with elevated levels of ALT should be referred to secondary care centers.

Conversely, a recent population-based cohort study demonstrated that all histological stages of NAFLD, including simple steatosis, are associated with a significantly increased risk of overall mortality that increase in a dose-dependent manner with increasing NAFLD severity^17^. Therefore, future research should focus on evaluating the prognosis of patients with MAFLD/NAFLD using the FAST score, which includes steatosis as a factor.

We previously questioned 334 outpatients regarding (1) the Alcohol Use Disorders Identification Test (AUDIT), a 10-item questionnaire designed by the WHO to screen for hazardous drinking in primary health care settings, and (2) frequency, type, and quantity of alcohol consumed^18^. We confirmed the daily alcohol consumption, calculated from frequency, type, and quantity as an accurate reflection of AUDIT^18^. In the Japanese health examination, two items are asked: frequency and quantity of alcohol consumption. Based on our studies, we believe that the method of calculating the quantity of alcohol consumption described in the method accurately reflects the quantity of daily alcohol consumption.

Several limitations must be considered when interpreting our study results. First, this was a retrospective, single-center study. Second, as mentioned, self-selection bias and referral bias were major limitations. Third, because this study was conducted at a health examination center, we used LSM instead of liver biopsy as a standard. Fourth, when using the LSM, technical failure was a common phenomenon (range, 6.7–27.0%) that was primarily related to high BMI^19,20^. During our analyses, approximately 20% of participants reported unreliable measurement results (data not shown). Although using an XL probe may be recommended for such patients, it was unavailable during the study period. Fifth, as we used data from a health examination registry, we do not have any information regarding liver biopsy. To confirm the findings of this study, further studies are needed that include biopsy-diagnosed NAFLD in patients with mild disease.

In conclusion, approximately 9% and 8% of the general Japanese patient population with MAFLD and NAFLD had a condition that could not be ruled out as progressive liver disease. Furthermore, the FLI, FIB-4, and ALT levels were independent determinants of progressive liver disease in patients with MAFLD.

The results of this study will provide useful information to general practitioners and clinicians who perform health examinations. They will also help in the selection of patients with MAFLD who should undergo further evaluations such as liver biopsies, resulting in a possible reduction in unnecessary interventional procedures.

## Methods

### Study design and population

We retrospectively enrolled participants for this cross-sectional study from the ongoing MedCity21 health examination registry between April 1, 2014 and December 31, 2019. This registry protocol is a comprehensive agreement. The study design was approved by the Ethical Committee of Osaka City University Graduate School of Medicine (approval no. 2927)^21^. Furthermore, this cross-sectional study of liver disease was part of the MedCity21 health examination registry and was conducted in full accordance with the tenets of the 1975 Declaration of Helsinki (6th revision, 2008). The study protocol was approved by the Ethical Committee of Osaka City University Graduate School of Medicine (approval no. 2019–076, February 21, 2020). The Ethical Committee of Osaka City University Graduate School of Medicine waived the need for written and verbal approved informed consent from the enrolled participants because this was a retrospective observational study using only existing information. Instead, we provided an opt-out option, explained in the instructions posted on the hospital’s website.

This study initially included participants (n = 3425) who had undergone a medical examination, including abdominal ultrasonography and LSM, for the first time during the aforementioned study period. The exclusion criteria were: lack of data regarding hepatitis B surface antigen (HBsAg)/anti-hepatitis C antibody (HCV-Ab) (n = 373); a success rate (SR) < 60% (n = 365); LSM with an interquartile range (IQR) or median > 30% (n = 181); positive serology results for HBsAg (n = 80): alcohol intake ≥ 60 g/day (n = 74); positive serology results for HCV-Ab (n = 41); lack of data regarding glycated hemoglobin (HbA1c) (n = 42); and lack of data regarding platelet counts (n = 15). After exclusion, a total of 2,254 participants were analyzed (Fig. 1).

### Clinical assessment

During the clinical review, we obtained the following data: BMI; WC; blood pressure. After an overnight fast, blood samples were collected and analyzed following standard laboratory procedures to determine the total bilirubin, aspartate transaminase (AST), ALT, albumin, GGT, total cholesterol, low-density lipoprotein cholesterol, high-density lipoprotein cholesterol (HDL-C), triglyceride (TG), creatinine, fasting plasma glucose, and HbA1c levels. Participants who had a fasting blood glucose ≥ 126 mg/dL, HbA1c ≥ 6.5%, or those on treatment for diabetes, were defined as T2DM^22^. Hypertension was defined as a systolic blood pressure ≥ 140 mmHg, diastolic blood pressure ≥ 90 mmHg, or those on treatment for hypertension^23^. Dyslipidemia was defined as serum TC levels ≥ 220 mg/dL and/or HDL-C levels < 40 mg/dL and/or TG levels ≥ 150 mg/dL, or those on treatment for dyslipidemia^24^.

The Lumipulse HBsAg and HCV assays (Fujirebio Inc., Tokyo, Japan) were used to assess the serological markers in the serum, including HBsAg and anti-HCV-Ab. The FLI, which is one of the scores important for diagnosing hepatic steatosis, was calculated using BMI, WC, and serum levels of TG and GGT as previously described^25^: FLI = e^y^/(1 + e^y^) × 100, where y = 0.953 × ln {TG (mg/dL)} + 0.139 × {BMI (kg/m^2^)} + 0.718 × ln {GGT (U/L)} + 0.053 × {WC (cm)} – 15.745.

The severity of liver fibrosis was assessed using two noninvasive fibrosis markers. The FIB-4 index and the NFS were computed using the available parameters^26,27^. The FAST score was calculated according to the following formula^10^: FAST = {exp (–1.65 + 1.07 × ln (LSM) + 2.66 × 10^–8^ × CAP^3^ – 63.3 × AST^–1^)}/{1 + exp (–1.65 + 1.07 × ln (LSM) + 2.66 × 10^–8^ × CAP^3^ – 63.3 × AST^–1^)}.

The results were divided into three categories: low-risk NASH zone (FAST score ≤ 0.35); indeterminate zone (0.35 < FAST score < 0.67); and high-risk NASH zone (FAST score ≥ 0.67)^10^. We defined a FAST score ≥ 0.35 as undeniable progressive disease.

### Alcohol intake screening and definition of alcoholic liver disease

Daily alcohol consumption was calculated in grams using our modified template^18^. We classified the frequency of alcohol intake into three categories: 1 day/week; 3 days/week; or every day. We also classified each participant’s average alcohol consumption into four categories: 10 g; 30 g; 50 g; or 70 g. The daily alcohol consumption (g/day) was calculated as follows: [(frequency of alcohol intake) × (average alcohol consumption (g)]/7. Habitual alcohol intake was defined as an intake of 1–59 g/day of alcohol.

### Diagnostic criteria and definition of the MAFLD and non-MAFLD groups

MAFLD was diagnosed via radiologically diagnosed hepatic steatosis (via abdominal ultrasound) and the presence of any one of the following three conditions: overweight/obesity, DM, or evidence of metabolic dysregulation. Metabolic dysregulation was defined as the presence of two or more of the following conditions: WC ≥ 90 cm for men and ≥ 80 cm for women; blood pressure ≥ 130/85 mmHg or ongoing specific antihypertensive drug treatment; TG level ≥ 150 mg/dL or ongoing specific hypolipidemic drug treatment; HDL-C level < 40 mg/dL for men and < 50 mg/L for women; and prediabetes (fasting glucose level of 100–125 mmol/L or HbA1c level of 5.7–6.4%)^7^. We did not use the homeostasis model assessment of the insulin resistance score and high-sensitivity C-reactive protein levels during this study. The non-MAFLD population comprised patients who did not fulfill the aforementioned conditions.

### NAFLD and non-NAFLD

NAFLD was assessed based on ultrasound evidence of FLD and the exclusion of secondary causes such as viral hepatitis and excessive alcohol consumption (≥ 30 g per day for males and 20 g per day for females)^2,3^. The non-NAFLD population comprised patients who did not meet the above conditions. The overlap population comprise patients who did not meet the both criteria of MAFLD and NAFLD.

### Abdominal ultrasound and assessment of disease severity

Fatty liver was diagnosed via abdominal ultrasonography using the Toshiba Aplio 500 device (Toshiba Medical Systems Corporation, Ohtawara, Japan). Abdominal ultrasonography was performed for the MedCity21 registry by experienced medical sonographers registered with the Japan Society of Ultrasonics in Medicine. Hepatic steatosis was semi-quantified according to the criteria described by Hamaguchi based on the presence of hepatorenal contrast, bright hepatic echoes, deep attenuation, and vessel blurring^28^.

### Vibration-controlled transient elastography

VCTE was performed using an M-probe device. The details of the technique and investigational procedure for the LSM have been described previously^29^. The CAP was also measured using VCTE to stage steatosis. The LSM was calculated using a proprietary algorithm based on the ultrasonic attenuation coefficient of the shear wave of VCTE, which is an estimate of the total ultrasonic attenuation at 3.5 MHz. Only VCTE measurements based on at least 10 valid images, SR ≥ 60%, and IQR/median < 30% were considered reliable and used for statistical analyses. We used an LSM cutoff of ≥ 6.7 kPa for significant fibrosis (fibrosis score ≥ 2) and of 9.8 kPa for advanced fibrosis (fibrosis score ≥ 3), which has been reported to identify Japanese patients with NAFLD^29^.

### Statistical analyses

Sensitivity and specificity, which reflect the probabilities of false-positive and false-negative assessments, respectively, were determined for the selected cutoff values. The AUROC curve was also calculated. The Youden index was used to identify the optimal cutoff points. A logistic model was applied with a FAST score ≥ 0.35 as the outcome adjusted for confounding factors (or patient characteristics). Independent variables were determined by the Youden index and included the following: age 45 years or older; BMI ≥ 26.7 kg/m^2^; WC ≥ 90.5 cm; alcohol intake ≥ 1.0 g/day; ALT level ≥ 42 U/L; FLI ≥ 63.9; FIB-4 ≥ 1.75; and NFS ≥  − 1.23. Data were expressed as odds ratios and 95% confidence intervals. The correlations between noninvasive blood test results were assessed using Spearman's rank correlation test, with p < 0.05 considered statistically significant. All statistical analyses were performed using JMP 13.0.0 software (SAS Institute Inc., Cary, NC, USA).

## Supplementary Information

Supplementary Information.

## Data Availability

The datasets generated during and/or analysed during the current study are available from the corresponding author on reasonable request.

## References

[1] Younossi ZM (2016). Global epidemiology of nonalcoholic fatty liver disease—Meta-analytic assessment of prevalence, incidence, and outcomes. Hepatology.

[2] Chalasani N (2018). The diagnosis and management of nonalcoholic fatty liver disease: Practice guidance from the American Association for the Study of Liver Diseases. Hepatology.

[3] European Association for the Study of the Liver (EASL), European Association for the Study of Diabetes (EASD), European Association for the Study of Obesity (EASO) (2016). EASL-EASD-EASO Clinical Practice Guidelines for the management of non-alcoholic fatty liver disease. J. Hepatol..

[4] Haldar D (2019). Outcomes of liver transplantation for non-alcoholic steatohepatitis: A European Liver Transplant Registry study. J. Hepatol..

[5] Singal AK (2016). Nonalcoholic steatohepatitis is the most rapidly growing indication for simultaneous liver kidney transplantation in the United States. Transplantation.

[6] Shiha G (2021). Redefining fatty liver disease: An international patient perspective. Lancet Gastroenterol. Hepatol..

[7] Eslam M (2020). A new definition for metabolic dysfunction-associated fatty liver disease: An international expert consensus statement. J. Hepatol..

[8] Neuschwander-Tetri BA (2020). Therapeutic landscape for NAFLD in 2020. Gastroenterology.

[9] Sumida Y (2020). Current and new pharmacotherapy options for non-alcoholic steatohepatitis. Expert Opin. Pharmacother..

[10] Newsome PN (2020). FibroScan-AST (FAST) score for the non-invasive identification of patients with non-alcoholic steatohepatitis with significant activity and fibrosis: A prospective derivation and global validation study. Lancet Gastroenterol. Hepatol..

[11] Oeda S (2020). Diagnostic accuracy of FibroScan-AST score to identify non-alcoholic steatohepatitis with significant activity and fibrosis in Japanese patients with non-alcoholic fatty liver disease: Comparison between M and XL probes. Hepatol. Res..

[12] Hofmann WP (2020). The Fatty Liver Assessment in Germany (FLAG) cohort study identifies large heterogeneity in NAFLD care. JHEP Rep..

[13] Puri P (2020). Use of FibroScan-AST score to stratify high-risk nonalcoholic steatohepatitis in US veterans. Clin. Gastroenterol. Hepatol..

[14] Moriya A (2015). Roles of alcohol consumption in fatty liver: A longitudinal study. J. Hepatol..

[15] Ratziu V (2016). Elafibranor, an agonist of the peroxisome proliferator-activated receptor-alpha and -delta, induces resolution of nonalcoholic steatohepatitis without fibrosis worsening. Gastroenterology.

[16] Friedman SL (2018). A randomized, placebo-controlled trial of cenicriviroc for treatment of nonalcoholic steatohepatitis with fibrosis. Hepatology.

[17] Simon TG (2020). Mortality in biopsy-confirmed nonalcoholic fatty liver disease: Results from a nationwide cohort. Gut.

[18] Fujii H (2016). The Alcohol Use Disorders Identification Test for Consumption (AUDIT-C) is more useful than pre-existing laboratory tests for predicting hazardous drinking: A cross-sectional study. BMC Public Health.

[19] Wong VW (2010). Diagnosis of fibrosis and cirrhosis using liver stiffness measurement in nonalcoholic fatty liver disease. Hepatology.

[20] Yoneda M (2020). Advances in ultrasound elastography for nonalcoholic fatty liver disease. J. Med. Ultrason..

[21] Yoshida S (2020). Association of plasma xanthine oxidoreductase activity with blood pressure affected by oxidative stress level: MedCity21 health examination registry. Sci. Rep..

[22] American Diabetes Association (2010). Diagnosis and classification of diabetes mellitus. Diabetes Care.

[23] Umemura S (2019). The Japanese Society of Hypertension guidelines for the management of hypertension (JSH 2019). Hypertens. Res..

[24] Teramoto T (2007). Diagnostic criteria for dyslipidemia. Executive summary of Japan Atherosclerosis Society (JAS) guideline for diagnosis and prevention of atherosclerotic cardiovascular diseases for Japanese. J. Atheroscler. Thromb..

[25] Bedogni G (2006). The Fatty Liver Index: A simple and accurate predictor of hepatic steatosis in the general population. BMC Gastroenterol..

[26] Sterling RK (2006). Development of a simple noninvasive index to predict significant fibrosis in patients with HIV/HCV coinfection. Hepatology.

[27] Angulo P (2007). The NAFLD fibrosis score: A noninvasive system that identifies liver fibrosis in patients with NAFLD. Hepatology.

[28] Hamaguchi M (2007). The severity of ultrasonographic findings in nonalcoholic fatty liver disease reflects the metabolic syndrome and visceral fat accumulation. Am. J. Gastroenterol..

[29] Yoneda M (2008). Noninvasive assessment of liver fibrosis by measurement of stiffness in patients with nonalcoholic fatty liver disease (NAFLD). Dig. Liver Dis..

