# Supplementary material for: The FibroScan-aspartate aminotransferase score can stratify the disease severity in a Japanese cohort with fatty liver diseases
Source: Sci Rep. 2021 Jul 5;11:13844. doi: 10.1038/s41598-021-93435-x (PMC8257719; doi:10.1038/s41598-021-93435-x)
Supplement: Supplementary file 1 — Supplementary Information. [file 41598_2021_93435_MOESM1_ESM.pptx]

## Slide 1
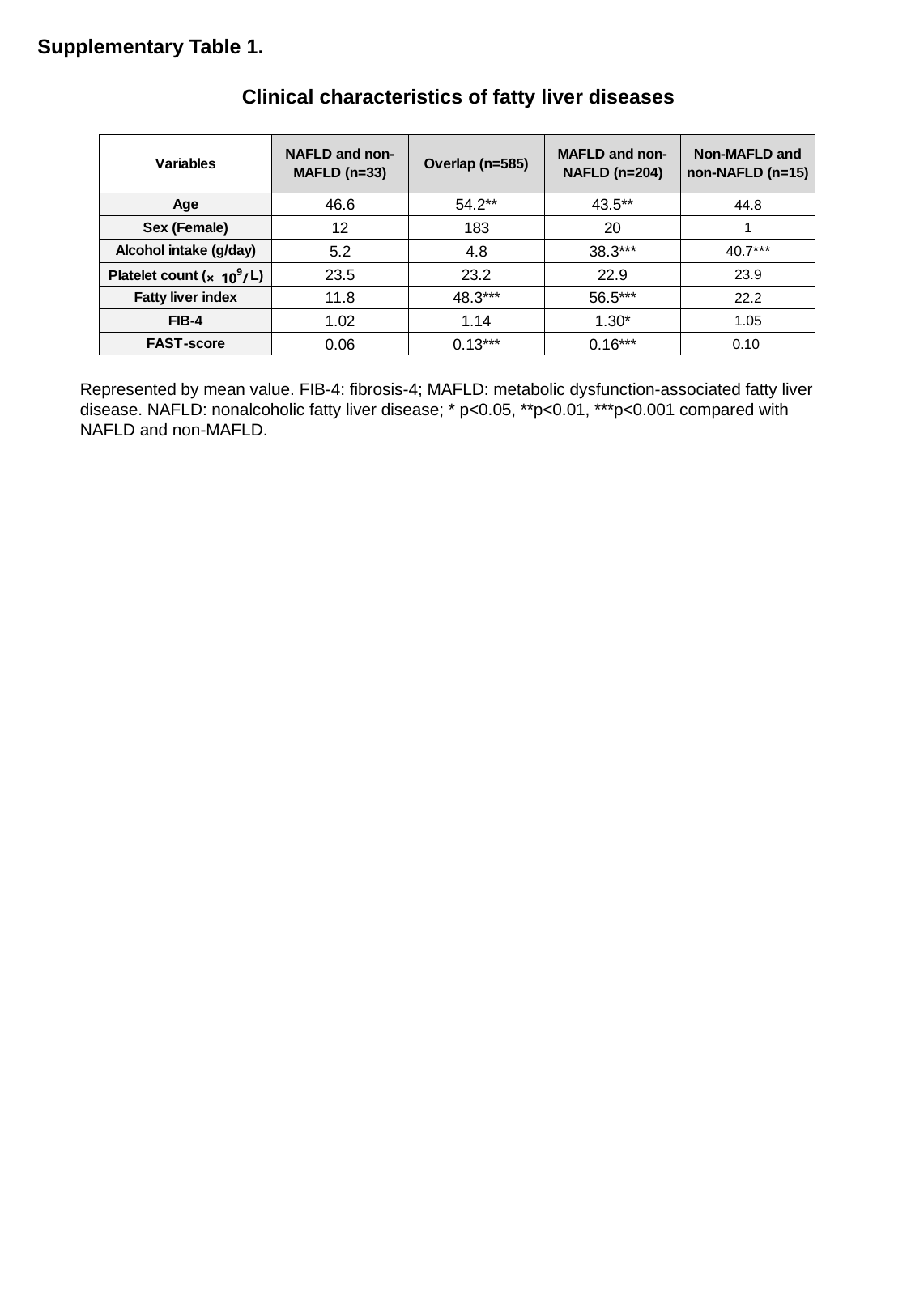

Supplementary Table 1.
Clinical characteristics of fatty liver diseases
Represented by mean value. FIB-4: fibrosis-4; MAFLD: metabolic dysfunction-associated fatty liver
disease. NAFLD: nonalcoholic fatty liver disease; * p<0.05, **p<0.01, ***p<0.001 compared with
NAFLD and non-MAFLD.

## Slide 2
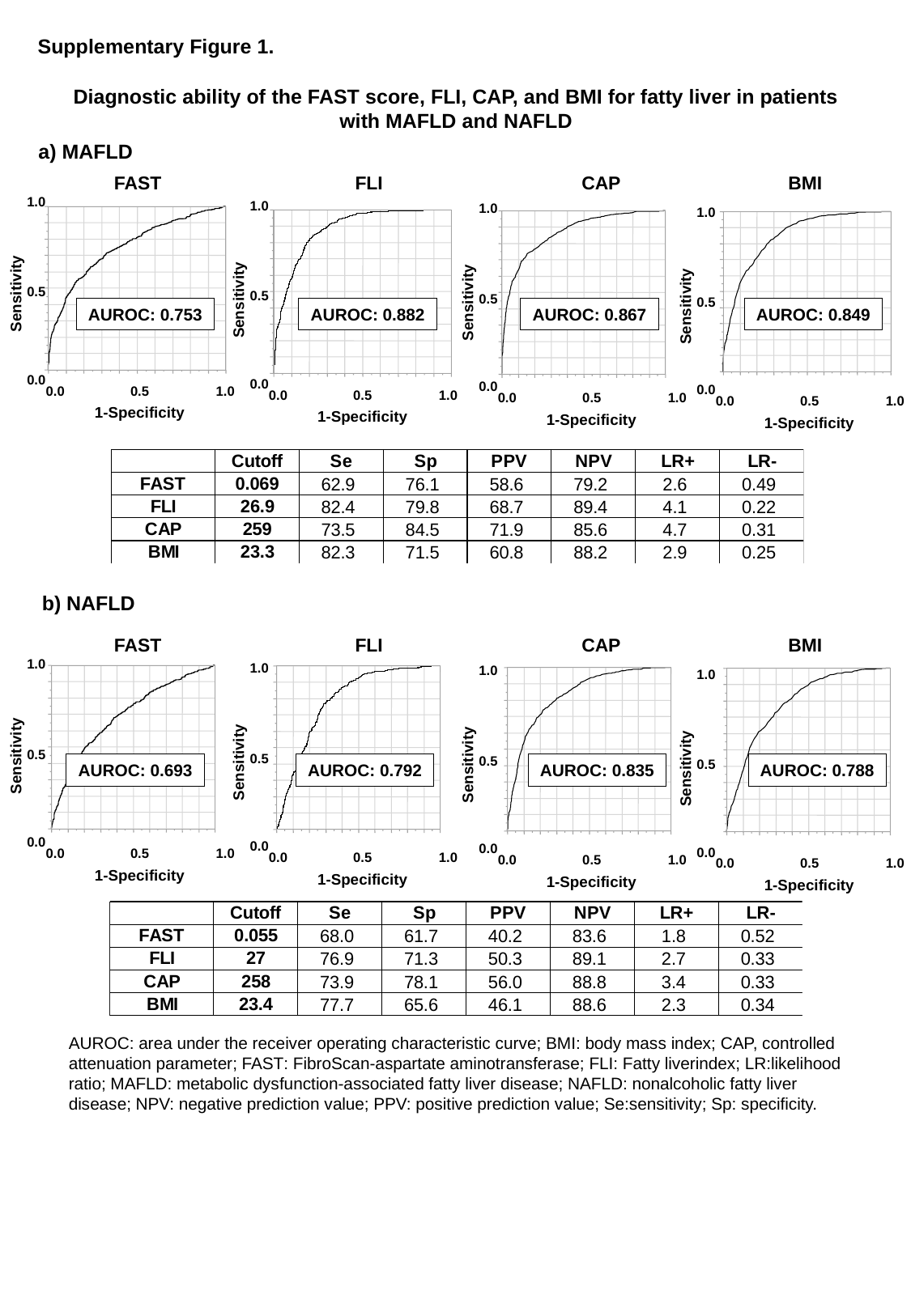

Supplementary Figure 1.
Diagnostic ability of the FAST score, FLI, CAP, and BMI for fatty liver in patients
with MAFLD and NAFLD
a) MAFLD
FAST
FLI
CAP
BMI
1.0
Sensitivity
0.5
0.0
0.0
0.5
1.0
1-Specificity
1.0
Sensitivity
0.5
0.0
0.0
0.5
1.0
1-Specificity
1.0
Sensitivity
0.5
0.0
0.0
0.5
1.0
1-Specificity
1.0
Sensitivity
0.5
0.0
0.0
0.5
1.0
1-Specificity
AUROC: 0.753
AUROC: 0.882
AUROC: 0.867
AUROC: 0.849
b) NAFLD
FAST
FLI
CAP
BMI
1.0
Sensitivity
0.5
0.0
0.0
0.5
1.0
1-Specificity
1.0
Sensitivity
0.5
0.0
0.0
0.5
1.0
1-Specificity
1.0
Sensitivity
0.5
0.0
0.0
0.5
1.0
1-Specificity
1.0
Sensitivity
0.5
0.0
0.0
0.5
1.0
1-Specificity
AUROC: 0.693
AUROC: 0.792
AUROC: 0.835
AUROC: 0.788
AUROC: area under the receiver operating characteristic curve; BMI: body mass index; CAP, controlled attenuation parameter; FAST: FibroScan-aspartate aminotransferase; FLI: Fatty liverindex; LR:likelihood ratio; MAFLD: metabolic dysfunction-associated fatty liver disease; NAFLD: nonalcoholic fatty liver disease; NPV: negative prediction value; PPV: positive prediction value; Se:sensitivity; Sp: specificity.

## Slide 3
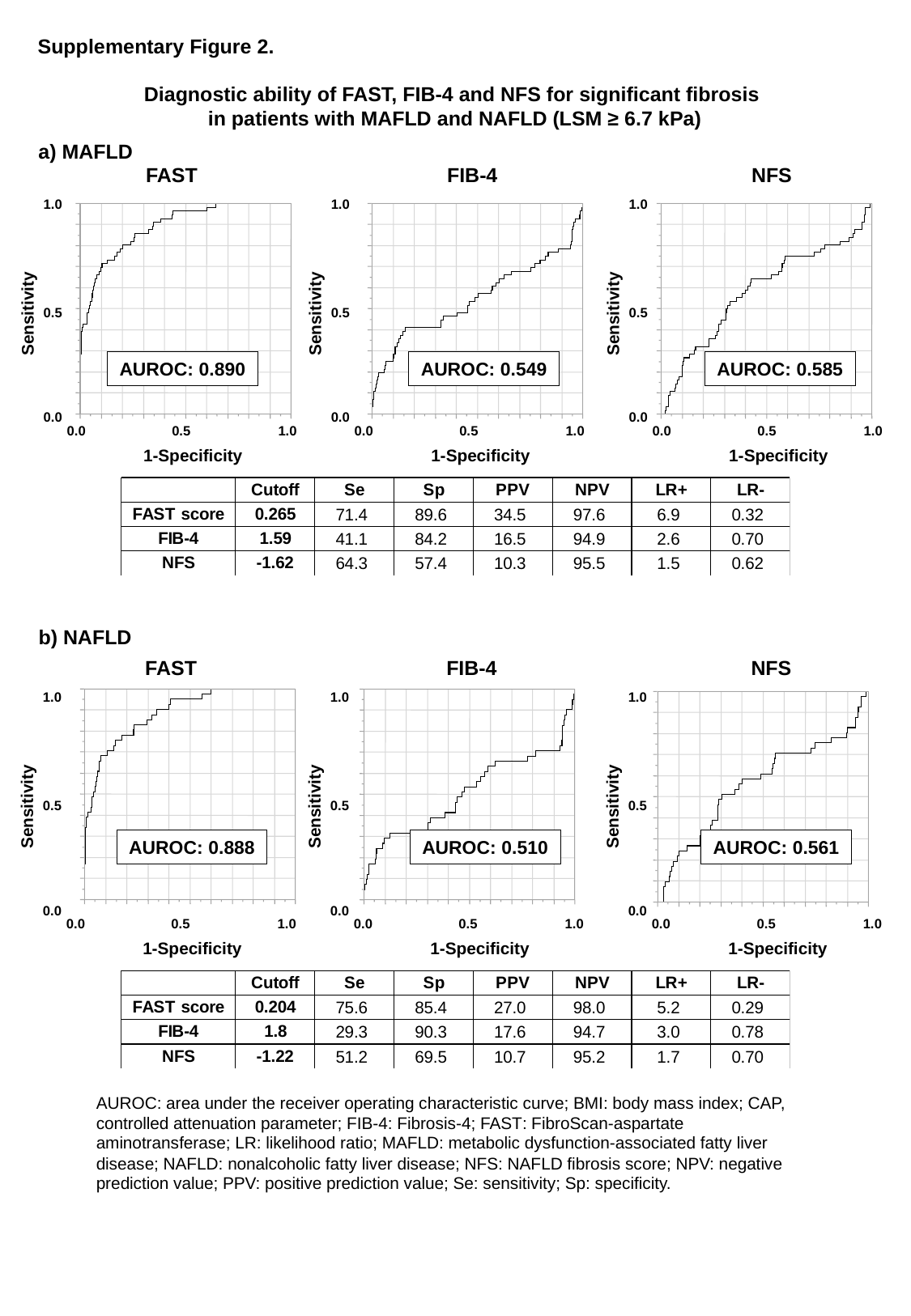

Supplementary Figure 2.
Diagnostic ability of FAST, FIB-4 and NFS for significant fibrosis
in patients with MAFLD and NAFLD (LSM ≥ 6.7 kPa)
a) MAFLD
FAST
NFS
FIB-4
1.0
Sensitivity
0.5
0.0
0.0
0.5
1.0
1-Specificity
1.0
Sensitivity
0.5
0.0
0.0
0.5
1.0
1-Specificity
1.0
Sensitivity
0.5
0.0
0.0
0.5
1.0
1-Specificity
AUROC: 0.890
AUROC: 0.549
AUROC: 0.585
b) NAFLD
FAST
NFS
FIB-4
1.0
Sensitivity
0.5
0.0
0.0
0.5
1.0
1-Specificity
1.0
Sensitivity
0.5
0.0
0.0
0.5
1.0
1-Specificity
1.0
Sensitivity
0.5
0.0
0.0
0.5
1.0
1-Specificity
AUROC: 0.888
AUROC: 0.510
AUROC: 0.561
AUROC: area under the receiver operating characteristic curve; BMI: body mass index; CAP, controlled attenuation parameter; FIB-4: Fibrosis-4; FAST: FibroScan-aspartate aminotransferase; LR: likelihood ratio; MAFLD: metabolic dysfunction-associated fatty liver disease; NAFLD: nonalcoholic fatty liver disease; NFS: NAFLD fibrosis score; NPV: negative prediction value; PPV: positive prediction value; Se: sensitivity; Sp: specificity.

## Slide 4
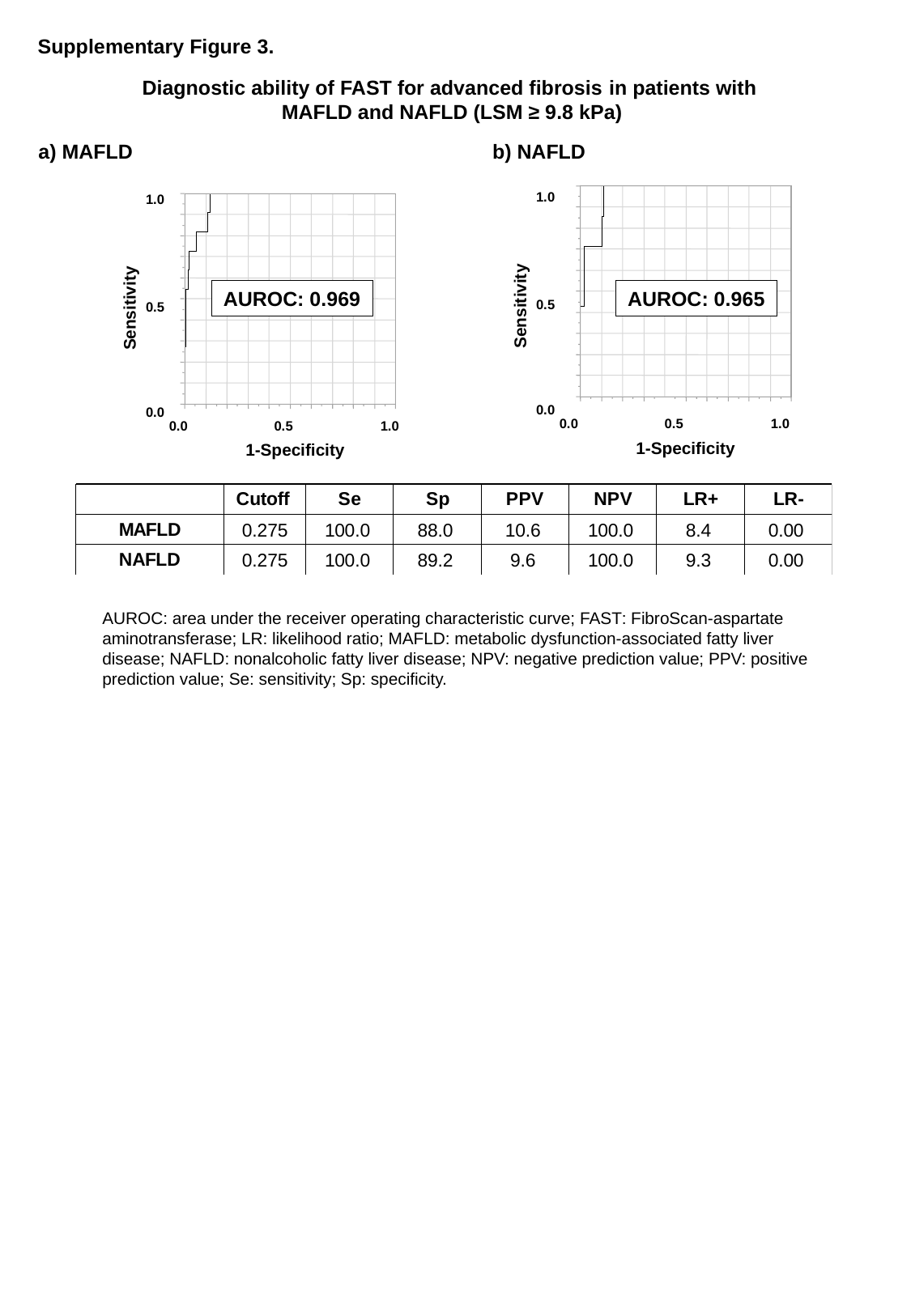

Supplementary Figure 3.
Diagnostic ability of FAST for advanced fibrosis in patients with
MAFLD and NAFLD (LSM ≥ 9.8 kPa)
b) NAFLD
a) MAFLD
1.0
Sensitivity
0.5
0.0
0.0
0.5
1.0
1-Specificity
1.0
Sensitivity
0.5
0.0
0.0
0.5
1.0
1-Specificity
AUROC: 0.969
AUROC: 0.965
AUROC: area under the receiver operating characteristic curve; FAST: FibroScan-aspartate aminotransferase; LR: likelihood ratio; MAFLD: metabolic dysfunction-associated fatty liver disease; NAFLD: nonalcoholic fatty liver disease; NPV: negative prediction value; PPV: positive prediction value; Se: sensitivity; Sp: specificity.

## Slide 5
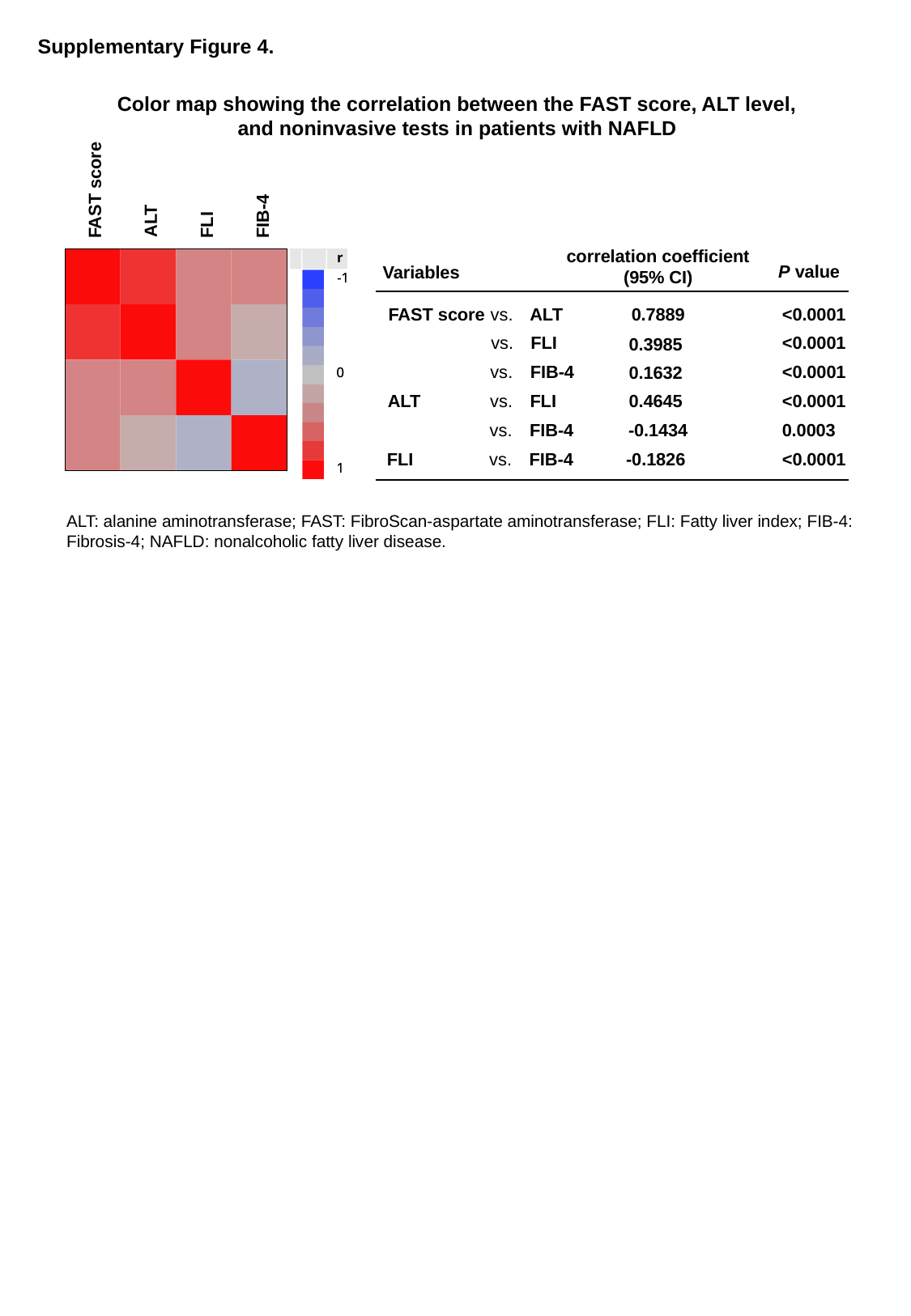

Supplementary Figure 4.
Color map showing the correlation between the FAST score, ALT level, and noninvasive tests in patients with NAFLD
FAST score
FIB-4
ALT
FLI
correlation coefficient
(95% CI)
r
r
P value
Variables
-1
-1
vs.
FAST score
ALT
0.7889
<0.0001
<0.0001
vs.
FLI
0.3985
<0.0001
vs.
FIB-4
0.1632
0
0
0.4645
<0.0001
vs.
ALT
FLI
0.0003
vs.
FIB-4
-0.1434
<0.0001
vs.
FLI
FIB-4
-0.1826
1
1
ALT: alanine aminotransferase; FAST: FibroScan-aspartate aminotransferase; FLI: Fatty liver index; FIB-4: Fibrosis-4; NAFLD: nonalcoholic fatty liver disease.
